# Acute Airway Obstruction and Cardiopulmonary Arrest due to Tracheomalacia Caused by Megaesophagus Compression Secondary to Achalasia

**DOI:** 10.1155/2020/5946985

**Published:** 2020-05-09

**Authors:** Mickael Aubignat, Pierre-Alexandre Roger, Amandine Dernoncourt, Valery Salle, Amar Smail, Clement Gourguechon, Jean Schmidt, Pierre Duhaut

**Affiliations:** ^1^Department of Internal Medicine, Amiens University Hospital, France; ^2^Department of Respiratory Diseases and Intensive Care Unit, Amiens University Hospital, France

## Abstract

We report the case of an 80-year-old woman who presented one episode of cardiopulmonary arrest and two episodes of acute airway obstruction. We found in this patient the presence of tracheomalacia caused by megaesophagus compression secondary to achalasia probably responsible for episodes of acute airway obstruction and cardiopulmonary arrest.

## 1. Introduction

Tracheomalacia (TM) is still an underdiagnosed disease but probably not a rare disease. In TM, we can find a reduction and/or an atrophy of longitudinal elastic fibers of the pars membranacea or impaired cartilage integrity. Prevalence in adults is unknown but is estimated between 12.7% and 44% from studies in selected populations [1, 2]. Bronchoscopy remains the golden standard for diagnosis of TM. It allows visualization of dynamic expiratory collapse [3, 4].

Achalasia (AL) is a neurodegenerative motility disorder of the esophagus that results in abnormal peristalsis and loss of lower esophageal sphincter function, especially during swallowing [5, 6]. Incidence is still rare, approximately 1.6 per 100,000 [7, 8]. Modalities utilized most frequently for diagnosis include endoscopy, radiographic studies, and manometry. Esophageal manometry remains the golden standard for diagnosis of AL with findings of aperistalsis and failure of relaxation of the lower esophageal sphincter [9, 10].

We report the case of an 80-year-old woman who presented one episode of cardiopulmonary arrest and two episodes of acute airway obstruction. We found in this patient the presence of TM caused by megaesophagus compression secondary to achalasia probably responsible for episodes of acute airway obstruction and cardiopulmonary arrest.

## 2. Case Presentation

An 80-year-old woman (46 kg, 154 cm) was transported to our intensive care unit intubated and ventilated with recovery of spontaneous circulation after cardiopulmonary arrest (no-flow 15 minutes, low-flow 10 minutes). She reported dyspnea and syncope in her toilets following a pushing effort. She had no previous history of respiratory or cardiac disease. She is followed for giant cell arteritis and myelodysplasia without other medical history.

**Figure 1:**
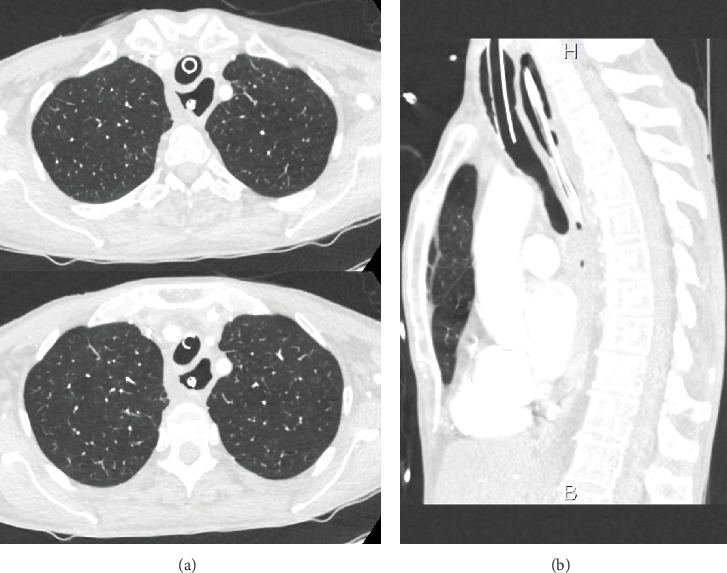
CT-chest showing megaesophagus with nasogastric tube and tracheal intubation but no tracheal compression. (a) Axial sections and (b) sagittal section.

Initial evolution was rapidly favorable, and the patient regained consciousness without sequelae. Etiologic assessment carried out initially did not explain this episode of cardiopulmonary arrest. CT-chest performed initially when the patient was intubated found a megaesophagus but no tracheal compression (Figure 1).

**Figure 2:**
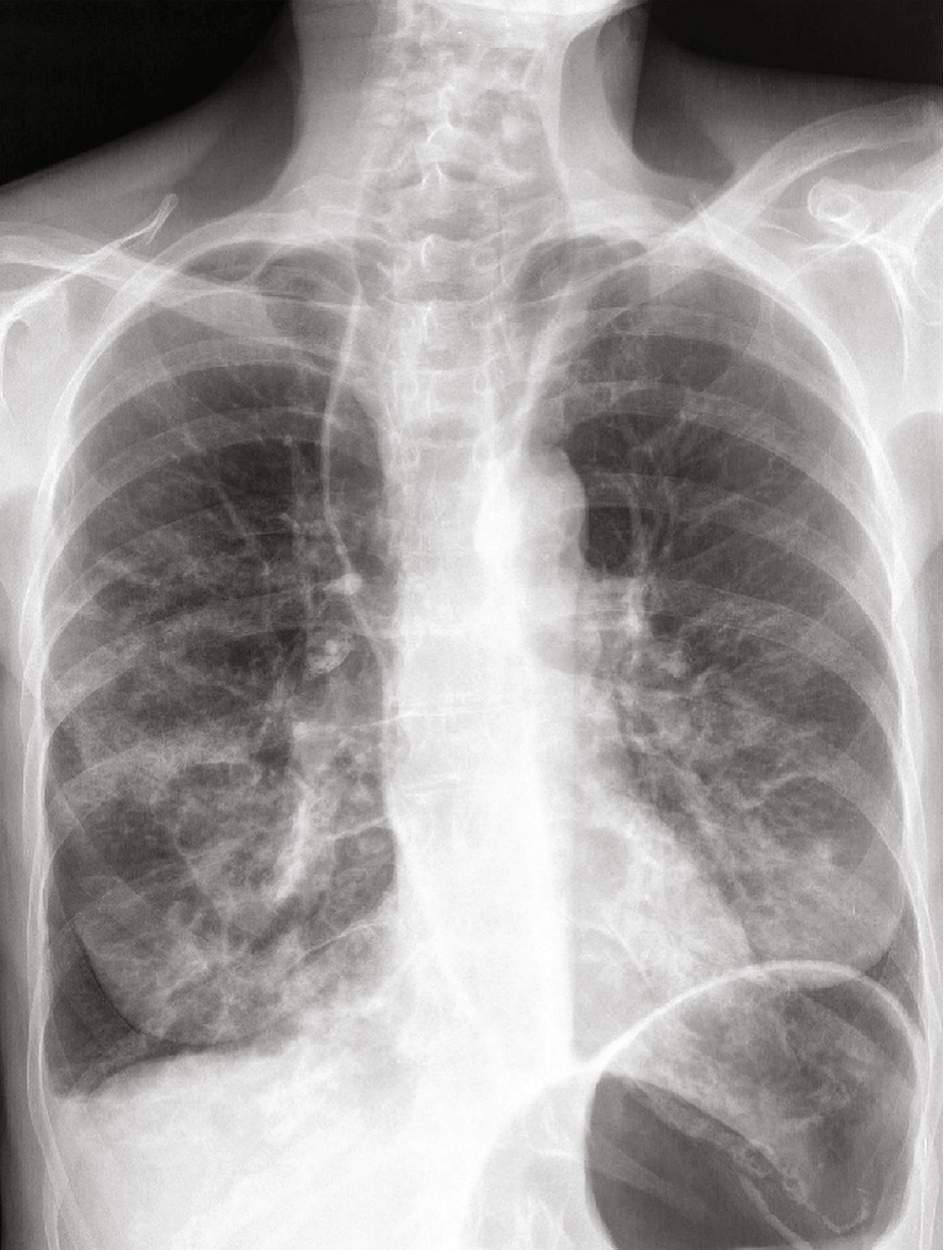
Anteroposterior chest X-ray showing enlargement of the upper mediastinum secondary to megaesophagus.

A week later, the patient presented an episode of acute respiratory distress with bradycardia during a meal. Faced by suspicion of inhalation pneumopathy, a bronchoscopy was performed but not finding of foreign bodies. In view of the presence of a pulmonary focus on chest X-ray (Figure 2), diagnosis of aspiration pneumonia was retained. The patient improved and returned home after few weeks.

**Figure 3:**
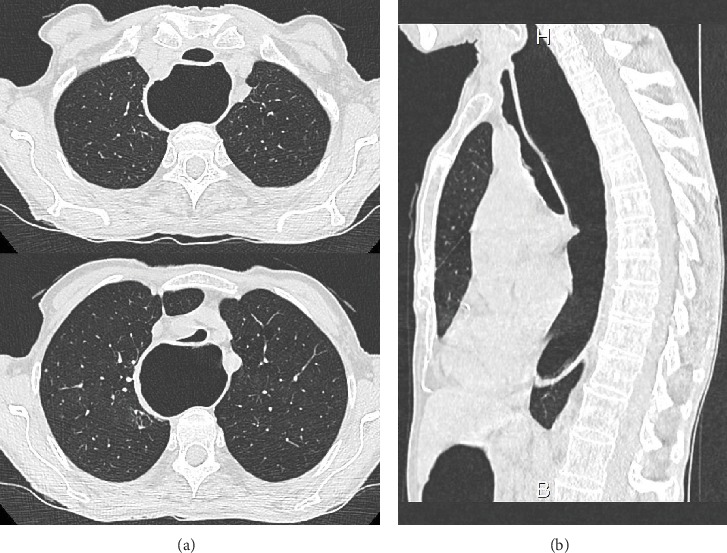
CT-chest showing megaesophagus with tracheal compression. (a) Axial sections and (b) sagittal section.

**Figure 4:**
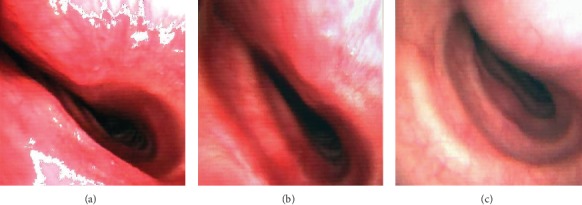
Trachea during bronchoscopic examination. (a) During expiration, at 10 cm from vocal cords with complete expiratory collapse. (b) During inspiration, at 10 cm from vocal cords with 80% inspiratory collapse. (c) Appearance of the trachea at the beginning of the tracheomalacia zone.

Three months later, the patient was rehospitalized in a state of acute respiratory distress, once again after a meal. At this time, new CT-chest without intubation found megaesophagus, this time with tracheal compression (Figure 3). Bronchoscopy performed at this time revealed a TM with complete expiratory collapse and 80% inspiratory collapse associated with an inflammatory mucosa (Figure 4).

In view of all these elements, we have retained the diagnosis of acute airway obstruction and cardiopulmonary arrest due to TM caused by megaesophagus compression secondary to achalasia. Achalasia was subsequently confirmed by esophageal manometry. No other cause of TM, in particular, no arguments for systemic infection or collagen vascular disease, was highlighted on various examinations. Please note that the patient's giant cell arteritis was perfectly controlled during these respiratory episodes.

On therapeutic plan, in front of patient advanced age and history of myelodysplasia with severe pancytopenia, surgery was not proposed. We proposed intraesophageal botulinum toxin injections under endoscopy to try to treat achalasia. The best treatment for TM is that of the cause. Following this treatment, the patient has not presented any cardiopulmonary arrest but she continued to have repeated lung infections. Unfortunately, she died two years later from a complicated lung infection.

## 3. Discussion

Bello et al. [11] first reported acute airway obstruction caused by achalasia in 1950. To date, approximately 40 such cases have been described. However, to our knowledge, only three cases of TM caused by megaesophagus compression secondary to achalasia [12–14] and a single case of cardiopulmonary arrest secondary to achalasia [15] have been described. Nevertheless, we can assume that cases of TM have been underestimated because in most cases of tracheal compression published, no bronchoscopy was carried out.

Acquired adult TM has many etiologies but is commonly idiopathic, posttraumatic, or secondary to chronic inflammation from recurrent infection and often presents in later life [16]. Symptoms may not be present until late in the disease process when degeneration of the tracheal cartilages is advanced. Presentation can often be confused with symptomatic asthma, and presentation is even more confusing by concomitant achalasia. Airway collapse results in changes in transtracheal pressure gradients that cannot be compensated by the weakened tracheal wall.

There are no well-defined recommendations for treatment of TM. Asymptomatic patients, in whom diagnosis of TM is fortuitously established, do not require treatment. In symptomatic patients, the first treatment is that of the cause. It is then possible to provide continuous positive airway pressure, tracheal prosthesis, laser treatment, or surgical management.
